# If things were simple, word would have gotten around. Can complexity science help us improve pediatric research?

**DOI:** 10.1038/s41390-024-03677-4

**Published:** 2024-11-28

**Authors:** Suzanne F. Fustolo-Gunnink, Willem P. de Boode, Olaf M. Dekkers, Gorm Greisen, Enrico Lopriore, Federica Russo

**Affiliations:** 1 Institute for Advanced Study, University of Amsterdam, Amsterdam, the Netherlands; 2 Department of Pediatrics, Division of Neonatology, Leiden University Medical Center, Willem-Alexander Children’s Hospital, Leiden, the Netherlands; 3 Sanquin Research & LAB Services, Sanquin Blood Supply Foundation, Amsterdam, the Netherlands; 4 Department of Neonatology, Radboud University Medical Center, Radboud Institute for Health Sciences, Amalia Children’s Hospital, Nijmegen, the Netherlands; 5 Department of Clinical Epidemiology, Leiden University Medical Center, Leiden, the Netherlands; 6 Department of Neonatology, Rigshospitalet and Copenhagen University, Copenhagen, Denmark; 7 Freudenthal Institute, Faculty of Science, Utrecht University, Utrecht, the Netherlands; 8 Department of Science and Technology Studies, University College London, London, UK

## Abstract

**Abstract:**

Complexity science is a discipline which explores how complex systems behave and how we interact with them. Though it is widely implemented outside medicine, particularly in the sciences involving human behavior, but also in the natural sciences such as physics and biology, there are only a few applications within medical research. We propose that complexity science can provide new and helpful perspectives on complex pediatric medical problems. It can help us better understand complex systems and develop ways to cope with their inherent unpredictabilities. In this article, we provide a brief introduction of complexity science, explore why many medical problems can be considered ‘complex’, and discuss how we can apply this perspective to pediatric research.

**Impact:**

- Current methods in pediatric research often focus on single mechanisms or interventions instead of systems, and tend to simplify complexity. This may not be appropriate.
- Complexity science provides a framework and a toolbox to better address complex problems.
- This review provides a starting point for the application of complexity science in pediatric research.

## Introduction

Complexity is a word we often use in common language to describe something that is difficult to understand. In complexity science, we make a distinction between *complicated* and *complex* systems. Complicated systems may have many parts that interact in intricate ways, but the system can be pulled apart, its components analyzed, the system understood and its future behavior predicted. An example is a car, where despite it being a complicated machine, we can predict what will happen when we change a part. In contrast, complex systems have certain characteristics, which we will discuss later, which make the whole more than the sum of its parts, and which make them to a certain extent unpredictable.^1–3^ Think for example of hurricane trajectory predictions, which, despite enormous amounts of data and on the clock analyses, are almost never 100% accurate. What characterizes hurricanes and makes them so difficult to predict? To what extent are health outcomes of children also unpredictable? And most importantly, how can we best deal with this unpredictability when doing research or when taking treatment decisions in clinical practice?^4^ (Box 1).

In this article, we will discuss the characteristics of complex systems and use clinical examples to show how many problems in pediatrics can be considered complex. Moreover, we will discuss how complexity science deals with this complexity and the resulting unpredictability, both in terms of research, as well as in terms of clinical management. Our thinking on how to apply the complexity perspective in pediatrics is still in its infancy. In this article, we intend to start the discussion on complexity in pediatrics, with the longer-term aim to develop research projects based on complexity science.

Box 1 Complexity in neonatal transfusion medicine A neonatal platelet transfusion trial published in 2019 showed increased risk of bleeding and/or mortality in infants treated according to a 50 × 10^9^/L versus 25 × 10^9^/L platelet count transfusion threshold. Long term neurodevelopmental outcomes were also worse in the 50 × 10^9^/L arm. Current clinical practice is highly variable, the mechanism of harm yet to be elucidated, and the optimal threshold still unknown, as thresholds lower than 25 × 10^9^/L have not yet been tested. Moreover, the optimal threshold is likely to be something else than a single platelet count number. There is evidence for biological developmental mismatch, as neonatal platelets differ from adult donor platelets, and increasing evidence showing non-hemostatic platelet functions including immunologic and inflammatory functions. The effects of donor- and transfusion product characteristics such as donor sex or storage time are unknown. And most importantly, the causal relationship between platelet count and bleeding risk is unclear, as platelet count alone does not seem to be a strong predictor for bleeding. Thrombocytopenia often occurs in the context of many other complications such as sepsis or necrotizing enterocolitis, and many other interventions such as mechanical ventilation and antibiotics. In the midst of this, clinicians are asked to make evidence-based transfusion decisions for infants with thrombocytopenia.^57–62^

## What is complexity science?

### Origins of complexity science

Complexity science originates from cross-disciplinary collaborations starting in the 1980s between computer scientists, physicists, chemists and biologists.^5^ These scientists realized that the systems they were looking at in their own fields, had striking similarities with systems in apparently unrelated fields. These systems varied from chemical reactions to biological processes to political systems and economies. They realized that these systems had similar characteristics and contained a certain level of inherent unpredictability and started to model this using sophisticated mathematical modeling strategies. Most of all, they realized the value of true interdisciplinary research to create a better understanding of complex systems. Over the years, many of the characteristics of complex systems have been defined, and complexity science has been applied in many fields, including physics, chemistry, biology, but also management and social sciences, and many research institutes on complex systems have been established worldwide.

### Characteristics of complex systems

In Table 1, we describe some of the key characteristics of complex systems.^3,5^ The first four characteristics are considered to be the essential conditions for complexity to arise and in complex systems, these conditions will give rise to some or all of the remaining six characteristics or products. For a system to qualify as a complex system, the initial four conditions plus the products self-organization and robustness must be present. The other characteristics are often present but are not critical.

**Table 1:** Characteristics of complex systems.

ESSENTIAL CHARACTERISTICS^a^		
Characteristic	Explanation	Examples in medicine
1. Numerosity	Complex systems involve many interactions among many components or agents	Molecules, cells, organs, humans, organizations or ecosystems.
2. Disorder and diversity	Interactions in complex systems are not coordinated centrally, occur randomly (disorder), and the agents may differ. Spontanenous order emerges as a result of random interactions.	Blood clots develop based on local interactions of many agents including platelets, red cells, clotting factors, endothelial cells etc. A decrease in order (or increase in randomness) is often a sign of a failing system, e.g., a decrease in fetal heart rate variability is a sign of fetal deterioration.^49^
3. Feedback	Interactions in complex systems are iterated so that there is feedback from previous interactions	Cerebrovascular reactivity in the neonatal brain is regulated via many different feedback loops, including increased intraluminal pressure, hypoxia, hypercapnia, hypoglycemia and neuronal activation. When cerebrovascular blood supply changes, this then changes the strength and directions of the feedback loops.^50–52^
4. Non-equilibrium	Complex systems are out of equilibrium, because they are open systems. This implies that there is a constant influx and loss of energy, and the systems are always on the verge of tipping out of balance, rather than being in a safe middle.	The human immune system is highly effective in eliminating a range of pathogens, but this is based on a delicate balance of activating and deactivating feedback loops. A relatively small change can derail this balance, leading to, for example, auto-immune responses.^53^
**RESULTING PRODUCTS**		
5. Spontaneous order and self-organization (emergence)	Complex systems exhibit structure and order that arise out of the random interactions among their parts.	Tumor development, clot formation and brain plasticity are all examples of how cells reorganize. Self-organization also occurs in for example social behaviors.
6. Non-linearity	Complex systems exhibit nonlinear dependence on parameters. This means that a small change in one parameter may lead to disproportionate changes in outcome and vice versa, in contrast to a proportional change as happens in linear relationships.(85) The system ‘amplifies’ it, so to speak.	Changes in body temperature within the normal range have little effect on our health but of higher or lower temperatures can be very detrimental. In the NICU, two infants may seem similar clinically, but small differences may ultimately result in vastly different clinical outcomes. Many biological processes are non-linear.^54–56^
7. Robustness	The structure and function of complex systems is stable under relevant perturbations. This means that the system can maintain its structure and function even if large changes happen.	Neonates can, in principle, recover from significant shifts in cardiorespiratory status while maintaining their core functions.
8. Nested structure and modularity	There may be multiple scales of structure, clustering and specialization of function in complex system. This means that systems consist of systems and are part of systems, and that some subsystems can be very specialized.	Systems within molecules are connected to systems within cells, which are connected to organ-level systems, etc.
9. History and memory	Complex systems often require a very long history to exist and store information about history.	For example, social systems, cities, etc. Clinical examples include the development of the immune response, which requires cumulative exposure to a range of pathogens. Hospital organizational structures develop over time.
10. Adaptive behavior	Complex systems can modify their behavior depending on the state of the environment and the predictions they make about it.	This can range from simple behavior of bacteria moving up a chemical gradient in search for nutrients to the most sophisticated human deliberation. Medical examples abound, for example: asymmetrical growth restriction, brain plasticity, but also adaptations in social systems.

^a^Based on Ladyman, J. & Wiesner, K. What Is a Complex System? (Yale University Press, 2020).

Self-organization or emergence requires further explanation. Emergence means that there is spontaneous order and organization as a result of many interactions between multiple parts. Not only does the system respond to its environment, but it is able to change its internal structure in response to it. For example, economies change their structure in response to money supply, growth rate, political stability, etc. The human brain changes its structure in response to things we do repeatedly – ‘neurons that fire together, wire together’. The architecture of a tumor varies depending on the supply of nutrients and oxygen and its interaction with immune cells, etc.^6^ Key requirement for emergence is that there is no a priori design, and no central control, emergence happens solely via interaction of the system with its environment through interactions among its parts.^3^

### Recognizing complex systems

There is little debate in the field of biology on the fact that (human) cells, organs and the (human) body are complex systems.^7,8^ Social, environmental, economic and many other systems are considered complex as well and there have been strong pleas for the use of a complexity approach in public health.^9,10^ In clinical medical research, we deal with many of these complex systems simultaneously, because we are interested in how a diagnosis or a treatment affects the (long term) health outcomes of children.^11–14^

How can we know whether something is a complex system? There is no such thing as a complexity-test. A system with many interacting parts that defies our abilities to understand or predict it, and that meets the criteria described in the table, should makes us wonder whether we should be studying such a system through the lenses and methods of complexity science. If there are no observable patterns at all (chaos), prediction is impossible and a complexity approach may not help. The questions we ask also matter. If we want to predict whether summers are warmer than winters, we may not need to consider complexity. If we want to predict the exact temperature on June 19, 2025, we do.

It is important to realize that we can look at complexity at many different levels. For example, we can look at a complex health issue within a cell, an organ, a human body, an intensive care unit, a hospital system, a national healthcare system, or on a global scale. Each of these are systems within systems, and each consist of many components and can qualify as a complex system, but they require different approaches and considerations. The level at which one approaches a system will depend on the questions one is trying to answer. In this paper, we focus on human health and clinical medical research, which involves several levels of complexity. We will pragmatically refer to *biological complexity* (everything that happens inside the human body, including the effects of interventions), *within hospital complexity* (everything that happens in the healthcare system setting, including interactions between parents, hospital staff, and collaborations with other centers) and *out of hospital complexity* (everything that happens between people inside and outside of the hospital, e.g., their social network, and everything that happens after patients are discharged, e.g., education) (Box 2).

We can also look at complexity over different timescales. This is important because the structure of a complex system can be more stable (e.g., a human cell) or less stable (e.g., an economy). We need to keep this in mind when we research complex systems. There is a time window within which we can expect stability, and therefore we can make good predictions of the system. But beyond that time window, predictions become very difficult. Different disciplines often look at different timescales with different methods.

Box 2 Acknowledging complexity before judging the clinical value of monitoring cerebral autoregulation A clinical example shows how several levels of complexity influence the decision whether to monitor cerebral oxygenation routinely in preterm infants, or not. Low cerebral oxygenation is associated with risk of severe intracranial hemorrhage and death. Cerebral oxygenation is affected by many different physiological factors simultaneously (numerosity, diversity), and these all interact at a local level (disorder) in various feedback loops (among others, autoregulation), and are affected by treatments such as vasoactive drugs, red blood cell transfusion and respiratory support. Many of the processes that determine cerebral oxygenation are non-linear. Furthermore, hypoxia-ischemia is only one pathway to brain injury, inflammation and metabolic stress are others (all adding to the *biological complexity*). On top of that, measuring cerebral oxygenation is imprecise and preterm infants are known to have a fragile clinical status. Preterm infants can get very sick, very quickly, the balance between under- and overtreatment is delicate, and clinicians differ in attitude (*within hospital complexity*). Most importantly, the patient-relevant outcome, i.e., the infant’s neurodevelopment will be influenced not only by these biological processes, treatment choices and potential brain injuries, but also by a myriad of other factors, including the infant’s social environment, parental education, financial resources, etc (out of hospital complexity).^50–52^

### Understanding complex systems

Complex systems, because of characteristics such as self-organization, non-linearity and adaptive behavior, are unpredictable, in the sense that we can never know with 100% certainty how the system will behave or what the effect of an intervention will be. This unpredictability is crucial for the system, as it reflects its adaptability, robustness, and self-organizing capability (Box 3).

In a sense, many systems can be considered complex, and they are all connected to other complex systems. Does that mean that everything is always unpredictable and we should give up on research? On the contrary. Complexity does not mean that everything is happening randomly and we cannot know anything. As described before, complex systems are unpredictable to some extent, but they can form patterns which we can analyze to better understand their behavior. This is where complexity science provides us with tools to identify these patterns and improve our understanding of complex systems. The key is to remember that because the patterns are formed by a complex system, any internal change or external intervention may cause that complex system to adapt or shift in ways we cannot always foresee, especially when we look at long term outcomes (Box 4). One important implication of complexity science is that it is unlikely that there is a single disease pathway or mechanism that we can intervene on to ‘shift’ the system completely, because that would make a system highly vulnerable. There are often collateral pathways, there is redundancy, and the system’s ability to adapt. Although there have been examples of successful single golden bullet treatments, such as vitamin C for scurvey, or surfactant for respiratory distress syndrome, these are most likely exceptions to the rule. A second implication is that when we intervene in a system, the system responds, and then we respond, so action and reaction are ‘entangled’, and co-evolve.

Box 3 The immune system as a complex system For example, Our immune response is very efficient and can cope with a wide range of pathogens effectively, exactly because there is no rigid central control and many processes happen simultaneously, locally, and in constant interaction. This lack of central control comes with the risk of the system going out of control, such as with auto immune disease. But if we imagine a central control only system for the immune response, which would need to first receive news of a newly located pathogen, then decide which immune cells to recruit, send them to the right location, and then hear back whether they were able to neutralize it, we can imagine this would be a very controlled, but slowed-down and probably inefficient system.

Box 4 Intervening in a complex system Another example of within-hospital complexity was the introduction of small-volume laboratory assays in the NICU of one of the authors. The expectation was that this would reduce blood loss due to blood draws. In contrast, the intervention initially increased the number of tests, most likely because clinicians justified extra diagnostic tests with the fact that they were only using small volume assays. The system adapted to the new situation, but not in the way that was expected. [unpublished data]

Diabetes type I at first seems like a simple problem: the body does not produce insulin, so we supplement the insulin. And given the fact that patients can closely monitor their glucose levels, we should be able to predict the effect of insulin at any given moment, and easily manage to keep glucose levels within acceptable ranges. However, extensive studies have shown that the reality is very different. And this, as Greenberg et al. explain very eloquently from both a patient’s and a scientist’s perspective, is because the problem is complex. ‘Blood sugar levels are not manufactured or produced by calculations and a treatment decision. Blood sugar levels are an ever-emergent property of many (unpredictable and unknown) processes in the body and the interactions (…) in the world, many of which are also unpredictable. (…) The good news is that while you can’t predict your blood sugar numbers, they are not random, and from the complex domain you can see patterns, or dispositions. Discovering these, you can influence your numbers in the right direction. Still, there is no certainty, so you cannot be held accountable for the results. Your effort becomes monitoring and knowing what to do when a blood sugar number appears on your meter. That’s the criterion for success.’^63,64^ The acceptance of complexity paves the way for more realistic expectations.

### Don’t we just need more data?

The most common argument against complexity science is that the unpredictability of complex systems results from a lack of knowledge. ‘We just don’t have enough data, but once we have more data, we will understand the system and eliminate unpredictability.’ Complexity science states that although lack of data is clearly a problem in medicine, because of the way complex systems function, some degree of unpredictability will always remain. So why exactly won’t collecting more data solve the problem of unpredictability in complex systems? We identify three main arguments.

1. The reference class problem. In clinical research, when everything is measured, every human being is unique and therefore different. To make accurate predictions, we would need to include reference groups in our studies for each of the relevant characteristics that can differ between people, and all the interactions and feedback loops. Evidently, there are too many variables and not enough data (and humans) in the world to solve this. This will mean that patients will always differ in some ways from patients in clinical studies, and therefore predictions will sometimes fail and treatments that are expected to benefit patients may not.^15,16^
2. Changes over time. Humans and their environment change constantly. The moment we finish a study, the world has already moved on and our predictions or understandings – even if they were impeccable at the time the data were generated - may be out of date. In addition, intervening in a system will often change the system. We are always a step behind; data cannot predict the future.
3. Interconnecting systems. Complex systems are often part of other systems and consist of subsystems. Most of the time, we cannot investigate all relevant systems that are connected to our system of interest. We need to put boundaries and because complex systems are open systems, change can always occur across these boundaries.

Some may argue that artificial intelligence will allow us to reduce unpredictability. Though we expect that artificial intelligence can improve our understanding of complex systems substantially, because it can account for the many interactions and feedback loops that conventional analytic methods cannot, we maintain our claim that there will still be irreducible uncertainty, for the same reasons as mentioned above.

## How can complexity science help us improve pediatric research?

Over the past years, complexity science has gained some traction in medical research, although, the focus has mostly been on health care organization, epidemiology and public health and not on applied clinical medical research.^9–12,14,17–23^ For various medical topics, there have been papers and books describing the need for a complexity approach, for example in sepsis, burn care, asthma, and related to the Covid pandemic.^11,17,24–27^ Several systematic reviews, which looked at applications of complexity science in health care, identified only 15–20 papers per year, of which only a minority of papers were about clinical research, and only a handful in pediatrics. Most importantly, these reviews highlight that although the concepts of complexity science are being embraced more and more, definitions vary, and practical applications are lacking. Some important advocates of complexity science within health care have highlighted this as well.^14,18,28–33^ In short, complexity science has not been explored sufficiently in pediatric research.

**Fig. 1:**
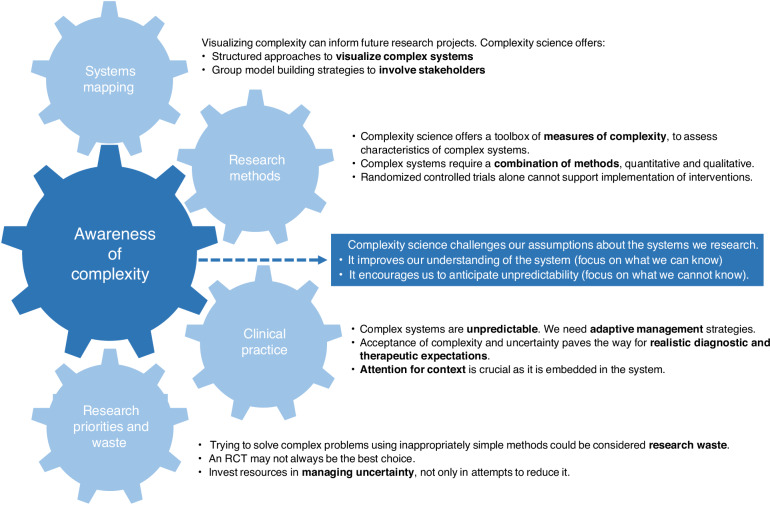
Conceptual framework of how complexity science could improve pediatric research and clinical practice.

We have identified several ways in which complexity science could improve pediatric research, which we will describe below, with accompanying examples. (Fig. 1) It is tempting to think that complexity science will provide us with ultimate solutions and magic bullets. However, if complex systems are truly as complex as we think they are, this is unlikely to happen. Instead, complexity science can provide us with better questions and a richer toolbox to start answering those.

### Complexity science is a tool to help us identify aspects of complex systems that we should address in research

In order to better understand a complex system, we need to look at it from as many angles as possible. Complexity science helps us be aware of the different characteristics of complex systems (e.g., self-organization, adaptation, feedback loops etc)^10,34,35^ (Box 5).

**Fig. 2:**
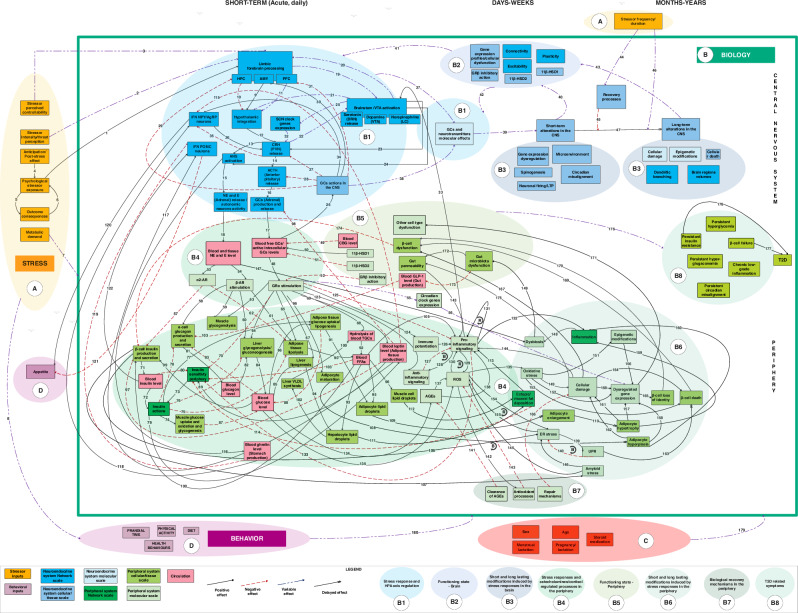
Causal-loop diagram (CLD) connecting processes involved in the stress response to the pathophysiology of T2D at different spatiotemporal scales. Reprinted from “How exposure to chronic stress contributes to the development of type 2 diabetes: A complexity science approach,” by N. Merabet, 2022, Frontiers in Neuroendocrinology volume 65, 100972. This is an open access article distributed under the terms of the Creative Commons CC-BY license, which permits unrestricted use, distribution, and reproduction in any medium, provided the original work is properly cited.

In addition, within complexity science, different methods for mapping or visualizing complex systems have been developed, which can help researchers get a better overview of many of the factors at play (Box 6, Fig. 2).

Box 5 Assessing different characteristics of complex systems The researchers of the recent BENEDUCTUS and PlaNeT-2/MATISSE neonatal clinical trials attributed some of their unexpected findings to biological complexity. In the BeNeDuctus trial it was postulated that higher rates of bronchopulmonary dysplasia (BPD) in preterm infants with a patent ductus arteriosus that were treated with the ibuprofen arm resulted from the unintended effect of ibuprofen on vascular growth factors in the developing lungs.^65^ And the increased risk of bleeding and mortality in the higher platelet count threshold arm in the PlaNeT-2/MATISSE trial was thought to result from non-hemostatic platelet functions.^57^ Perhaps these non-intended and unexpected effects could have been identified in advance with more thorough collaboration with biomedical researchers. But it is also possible that some of these unexpected effects are very difficult to predict, given the complexity of the developing human body. In any case, these examples highlight the fact that when we intervene in a complex system such as the human body, we can expect and should anticipate unexpected and unintended effects. ‘The only certainty is that there is no certainty.’^4^

Leykum et al. evaluated the way patients were discussed during rounds in an acute care facility, by looking at the way the rounds were organized and how patient outcomes were discussed. They found that these factors affected patient outcomes in terms of duration of stay and complication rates.^66^ These are social/behavioral factors that are not often assessed when looking at hard patient outcomes like complication rates. But a complexity perspective can remind us to look beyond what is immediately obvious.

Box 6 Mapping and visualizing complex systems Merabet et al. developed a causal loop diagram to visualize all the factors and interactions at play in the interaction between stress and diabetes^23^ (Fig. 2). A similar approach was used to map biological complexity related to drivers of instant local tissue oxygenation.^50^ This technique yields very complex and crowded (yet still incomplete) models. Though this may not seem helpful in clinical practice, it is most likely a better representation of the true complexity of the system. Practically, this and other systems mapping approaches can help researchers identify potential interactions in advance, identify leverage points (see “Complexity science can help us identify and avoid research waste and better prioritize research”) for interventions, or research gaps that need to be addressed.^23,36,67^

### Different aspects of complex systems will need different research approaches (measures of complexity)

**Fig. 3:**
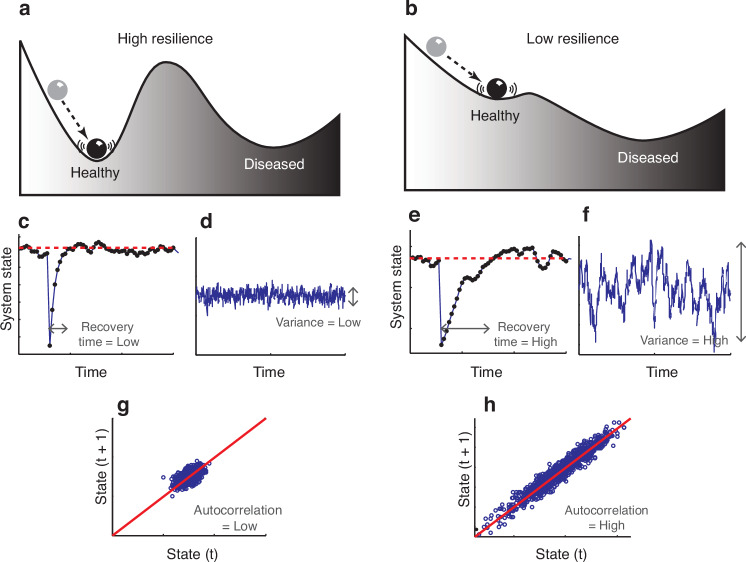
Mathematical model of homeostatic dynamics of a patient approaching a tipping point. Critical slowing down is a generic indicator that the patient has lost resilience in the sense that the patient may shift more easily from his current “healthy” state into an alternative “diseased” state. The patient (represented by the ball in the stability landscape in (**a**) and (**b**) can be in a diseased state (e.g., in a major generalized depression) and in a more healthy state (e.g., in a healthy mood state). Far from the tipping point, the patient is highly resilient, and perturbations will not easily flip the subject out of the basin of attraction to the alternative diseased state (**a**). Changes in conditions (e.g., drug application, stress, and comorbidity) can lower resilience and shrink the basin of attraction (**b**), so that a perturbation can more easily flip the patient into the severe disease state. As the basin of attraction becomes smaller, its slopes become less steep, implying that the return rate to equilibrium upon small perturbations slows down. Recovery time after a small perturbation is higher (E vs C) if the patient is closer to the tipping point. The effect of this slowing down can also be measured as (F vs D) increased variance in randomly induced fluctuations in the current state of the patient, caused by small external stressors, and (H vs G) in increased “memory” or increased (lag-1) autocorrelation between the serial measurements when the disease state is moving toward its tipping point. Reprinted from “Slowing Down of Recovery as Generic Risk Marker for Acute Severity Transitions in Chronic Diseases,” by M.G.M. Olde-Rikkert, 2016, Critical Care Medicine, Mar;44(3):601-6. Copyright © 2016 by the Society of Critical Care Medicine and Wolters Kluwer Health, Inc. All Rights Reserved. Reprinted with permission.

The different characteristics of complex systems, such as non-linearity, emergence, and feedback, cannot be measured using just one method but require a combination of so called ‘measures of complexity.’ Many measures of complexity have been introduced and the list is still growing. They vary from simple analyses such as counts and variance for numerosity and diversity to methods that require more sophisticated computational modeling, such as Shannon entropy, agent based modeling or power laws^5^ (Box 7, Fig. 3). Stronks et al. describe that within epidemiological research, we need to look at patterns in place, time, and on a person-level, interactions, non-linearity, feedback loops, adaptation over time, and many other aspects, each of which require different types of data and different analyses.^35^ For medical research, we have the additional challenging task to connect these data with biological complex systems data.

Computational, mathematical strategies can help to improve the way we describe and visualize complex systems, because they can incorporate many components and interactions.^36^ It goes beyond the scope of this article to describe them in detail, but we refer to reviews published elsewhere.^5^ Rod et al. provide a thorough overview of epidemiologic strategies, which includes items like life course models, network analysis, computer simulation models such as system dynamics models and agent-based models, functional data analyses, flexible AI based methods, etc^10^ (Box 8). Even though these methods may often feel like a black box, and come with their own challenges and limitations, we may need to accept that difficult questions about complex systems may require difficult methods. The alternative, trying to answer difficult questions about complex systems using only simple methods, will almost invariably lead to research waste and may potentially be harmful.

Importantly, complexity science challenges the status of randomized clinical trials as the single gold standard for medical treatment effects. Although there is a clear rationale for randomized controlled trials, and they are a valuable research tool, we should not forget that they only answer very narrowly defined research questions and attempt to isolate the effect of a single causal factor without looking at context and the system as a whole.

Aside from the known limitations of RCTs, complexity science adds the understanding that any treatment effect is in a sense a dynamic, emergent effect.^37,38^ If an RCT shows beneficial effect of treatment A, this effect may have occurred via different ‘routes’ within a complex system, such as interactions with patient characteristics or co-treatment, but also social factors such as decision making strategies or power dynamics, which may all change over time and vary between hospitals, patients and caregivers. In other words, the effect of an intervention depends on its context, though of course this dependency will vary depending on the type of intervention studied. The fundamental generalizability assumption of Evidence-Based Medicine is that as long as we copy the intervention in the same clinical population (following the RCT inclusion and exclusion criteria), we can expect a similar treatment effect (on a group level) in other hospitals. The underlying assumption is that context will be relatively similar or will have little impact on the effect. However, RCTs often do not provide us with sufficient information to ascertain whether the complex system in our hospital resembles the complex system in the RCT enough to warrant implementation of the intervention. The need for thorough considerations and lack of consensus on how to assess generalizability of RCTs has also been highlighted in epidemiologic literature.^39^ Therefore, though RCTs can be helpful in identifying potential treatment effects, complexity science claims that RCTs alone provide insufficient information to guide implementation in clinical care, and need to be complemented with other types of research as well as clinical judgement. Evidence-based medicine traditionally teaches an evidence pyramid, with systematic reviews and then randomized trials set at the top by default. Complexity science, on the contrary, sees different methods as complementary and interdependent, each answering related research questions, which are all needed to come to a fuller understanding of a system.^40^

Complexity science also places a strong emphasis on the human factor and social sciences research. People often don’t act the way we expect them to act. Patients, caregivers, nurses, and clinicians may have motivations, biases and unconscious convictions that impact the way they make decisions or deliver care. All these factors are part of the same complex system in which we implement medical interventions. An example from neonatology was a neonatologist who transfused a child at the high threshold during the Planet-2/MATISSE RCT in an infant randomized to the low threshold, because she had followed the protocol for the low threshold on the previous day and that child passed away due to bleeding. Thus, the personal experience of health workers is filtered through knowledge and belief systems to influence adherence to protocols and guidelines. Despite knowing that the bleeding was unlikely to be caused by withholding the transfusion, she could not make herself withhold another transfusion in a similar scenario. The psychological side of decision making, how healthcare workers cope with uncertainty, and many other factors are important and need to be taken into account when we consider diagnostic tools or treatments.^41^ This is also why qualitative research is important and highly relevant. Not everything can be captured in numbers. Qualitative research needs to be reinstated as a valid scientific method in medicine^42^ (Box 9).

Box 7 Example of measure of complexity for robustness Robustness refers to the ability of complex systems to resist perturbations (Fig. 3). Highly robust systems will ‘veer back’ from perturbations to their original state. However, with decreasing robustness, a complex system will at some point reach a tipping point, after which irreversible change towards another state will occur. This concept is helpful when we consider sudden clinical deterioration in the intensive care setting. Robustness can be analyzed by looking at critical slowing down: when a system is close to a tipping point, the time to recovery from perturbations is increased. Mathematically, vicinity to a tipping point can be measured by analyzing the functional dependency of the recovery time on the perturbation strength. We can also assess increases in fluctuations as a signature of a nearby tipping point. These measures of robustness of complex systems have been explored for several medical conditions, and could be of interest for, for example, pediatric or neonatal intensive care settings.^68^

Box 8 Computational strategies to model complex systems Nested structure, one of the characteristics of complex systems, means that structure in a system is repeated again on a smaller scale. The structure is nested within itself. This is also called scale invariance. A measure of complexity to assess this is the fractal. Fractals are complex patterns of self-similarity, such as we see in a cauliflower: when you break it into smaller parts, each part again resembles the larger cauliflower. Fractal patterns are ubiquitous in nature and a change in these patterns can signify a change in a system before more ‘simple’ variables such as heart rate start changing.^56^ Captur et al. describe how fractal analysis can be used in cardiology.^69^ Schnettler et al. analyzed other types of complex patterns in fetal heart rate analyses to predict intrauterine fetal demise (IUFD).^49^

Cognitive decline in Alzheimer’s disease is driven by various interlinking causal factors. Uleman et al. developed a systems dynamic model with 33 factors and 148 causal links and simulated the effects of interventions on outcomes as found in clinical trials and observational data. This suggests that computational modeling can be a helpful additional tool to assess the effects of interventions via simulation.^70^

Box 9 Different research methods to assess complex systems Videoreflective methods can be used to visualize the complexity of clinical care, and to form a starting point for reflection, learning, and better clinical practice. The advantage over methods such as simulations and debriefings, is that it allows recording of the unexpected events and nuances that occur in real-life situations, i.e., the ‘real complexity’ that is incredibly difficult to simulate.^71,72^

Sensemaking is the cognitive process by which we structure the unknown, understand and explain the world, and inform action. It is influenced by culture, prevailing narratives, experiences and knowledge systems. Understanding how sensemaking happens in the hospital setting, can help researchers and policymakers discover strategies to change or improve practice. It can also highlight areas of apophenia, defined as perceiving meaning in unrelated phenomena.^73^ Sensemaking in the hospital setting can be assessed using quantitative and qualitative methods, such as micronarratives: short descriptions of events that have happened in relation to a particular theme. These methods have been used to identify differences in sensemaking between nurses and clinicians in an emergency care setting.^74,75^

The design of randomized trials can be improved to, for example, allow for adjustments based on context or treatment effect. Examples include adaptive designs, SMART trials (sequential multiple assignment randomized trials), n-of-1 trials, and hybrid effectiveness-implementation designs).

### Complex systems require other types of interventions and strategies to manage them than complicated systems

We suspect that many clinicians may recognize the inherent unpredictability of the complex systems they are dealing with, but they may have come to accept that the only way to do research and to develop guidelines is by ignoring some of the complexity and simplifying the system. In fact, one could say that there is a mismatch: we perform research as if the systems we look at were complicated, even though in fact they are complex. Complexity science proposes another, more pragmatic approach, where we anticipate unpredictability, and develop treatment plans and a health care system that can cope with unexpected changes and can quickly adapt and improve.^18,43^ We can imagine that guidelines developed through the application of complexity science will be different, and will include strategies that allow for more flexibility in patient management.^12,13^ Of course this is a delicate balance because the stakes are high, and junior doctors especially need guidance to support their practice. This is a widespread difficulty across clinical practices, and needs further investigation in an interdisciplinary way.^13^ In this context, we propose that we can learn a lot from other equally complex disciplines and fields where complexity science is being applied (Box 10).

Box 10 Managing complex systems Greenberg et al. describe how complexity science has helped them gain a new perspective on managing diabetes type I (as described in Box 4). ‘The realization that living with type I Diabetes and managing blood sugar is primarily complex, not complicated, changed our thinking, our actions and lifted our emotional burden. The implications for healthcare professionals is that there are more effective approaches that can expand their repertoire in working with people with type 1 diabetes.’^63^

Simpkin et al. describe the need for a shift toward the acknowledgment and acceptance of uncertainty in the medical profession. They state this would benefit the mental health of health care professionals, the quality of the diagnostic process, and patient involvement in the decision-making process. ‘Medicine is a science of uncertainty and an art of probability. Ironically, only uncertainty is a sure thing. Certainty is an illusion.^4,76^

Reed et al. developed simple rules to guide translation of evidence into healthcare practice. Simple rules are heuristics, or mental shortcuts that allow people to make fast decisions that may not be perfect but are sufficient for reaching an immediate, short-term goal. These can help guide action in complex situations. For example, a simple rule from the US marine when battlefield command breaks down is ‘capture the high ground, stay in touch, keep moving.’ Simple rules have shown to outperform extensive guidelines in complex situations. As such, they may also be useful in clinical practice. Examples of the simple rules developed in this evidence translation project are ‘understand practices and processes of care’, and ‘facilitate dialog.^29,77^

When Michigan State University designed their new campus, rather than predicting where people would walk and placing a pavement accordingly, they put grass everywhere. They then intentionally allowed ‘elephant paths’ to emerge. One year later, they asphalted and formalized the pedestrian infrastructure.^78^ In this example, the designers realized they would not be able to predict how the complex system of campus inhabitants would behave. Instead, they opted to ‘probe’ and then act upon the actual behavior of the system.

### Complexity science is a tool to help us identify leverage points in complex systems

Leverage points are points in a complex system where a relatively small change could lead to a ‘shift’ or large change in the system’s behavior. Therefore, they are potential targets for interventions. Often, multiple interventions and leverage points need to be addressed to obtain change.^44,45^ Nobles et al. developed the Action Scales Model, a conceptual tool to identify key points for action within complex adaptive systems. They divided leverage points into events, structures, goals and beliefs. Intervening at the goals or beliefs level (e.g., changing people’s perception of unhealthy food) is often more effective than intervening at structural or event level (e.g., removing a snack bar from a neighborhood).^45^ In the medical context, addressing beliefs of clinicians regarding the effectivity of a certain treatment may be more effective than implementing a guideline requiring them to change their behavior. To our knowledge, little has been published on leverage points in relation to biological complexity. But we can imagine this approach will help identify new biological leverage points or a combination of leverage points which can be targeted (Box 11).

Box 11 Where to intervene in a complex system (leverage points) Implementation science focuses on what is needed to implement evidence into clinical practice. Initially, simple step-by-step approaches were used (the ‘pipeline’ models), but there is increasing awareness that this may not be effective in complex systems. Braithwaite et al. describe how the concepts of complexity science can help improve implementation science, using as an example the implementation of a rapid response system (RRS) in the Australian healthcare system. Factors that had to be addressed in that implementation process were clinician’s fears of more paperwork, the attitude that a patient was ‘owned’ by their admitting doctor, medical hierarchies, and onerous bureaucracy. At the same time, clinicians started to implement RRSs without active implementation mechanisms. There was a combination of bottom up and top-down activities. The tipping point for implementation of a large scale program was the preventable death of a teenager caused by failure to recognize the teenager’s deteriorating condition. In short, the process was nothing like a simple, linear, step-by-step program.^79^

The UK Medical Research Council’s guidance for developing and evaluating complex interventions has recently been revised and now includes explicit discussions on complexity concepts such as emergence and self-organization. ‘The framework challenges the view that unbiased estimates of effectiveness are the cardinal goal of evaluation. It asserts that improving theories and understanding how interventions contribute to change, including how they interact with their context and wider dynamic systems, is an equally important goal. For some complex intervention research problems, an efficacy or effectiveness perspective will be the optimal approach, and a randomized controlled trial will provide the best design to achieve an unbiased estimate. For others, alternative perspectives and designs might work better, or might be the only way to generate new knowledge to reduce decision maker uncertainty’.^80^

### Complexity science can help us identify and avoid research waste and better prioritize research

A system is not either complex or not complex, but shows variation in the degree of complexity, and our understanding of the system varies too. Sometimes we can identify patterns that can help us guide practice, and we improve our understanding of the system. Complexity science can help us distinguish between questions and methods that have the potential to contribute to this understanding, and those that don’t. For example, looking at a single intervention and a single outcome in an explicitly complex system will most likely not add to our understanding of that system, or help us in decision making, and could therefore be considered research waste. Complexity science helps us to assess the extent to which we need to take complexity into account, and make intentional and explicit decisions about this. If the patterns are quite clear, and stable, we have a lot of understanding of the system, and if not many other systems are involved, it may be entirely justifiable to use conventional methods to explore these patterns. But if such assumptions don’t hold, using conventional methods (even if applied correctly and with high quality data) will often lead to research waste (Box 12).

It is not a single study, but a combination of studies exploring complexity around a single theme to create a ‘thick causal story’, that we consider a hallmark for complexity inspired clinical research. We acknowledge that, particularly in pediatrics, there is a need for more research funding, support and resources, and this will impact the extent to which researchers and research groups are able to explore many aspects of a complex problem. But on the other hand, if complexity science helps us to focus our limited resources on the research questions that matter and can be answered, this may ultimately be a more efficient strategy.

Box 12 Research waste and prioritization Research waste is recognized as a pervasive and urgent problem in medical research. It can be caused not only by inappropriate design or insufficient reporting, but also by not asking the right research questions.^81,82^

‘In complex, chronic diseases such as diabetes, coronary artery disease, or asthma, a single factor is rarely implicated as solely responsible for disease development or presentation. Rather, multiple factors are often identified, and the disease evolves through complex interactions between them. Consequently, a perspective in which the interactions and dynamics are centrally integrated into the analytical methods may be better suited. Systems perspectives, unlike reductionisms, focus on these interrelationships and therefore may be the optimal method for complex chronic diseases.^83,84^

In pediatric research, we often deal with the reality of flawed, uncertain, proximate and sparse (FUPS) data. Sometimes this is the only data we have to inform practice. Wolpert et al. developed complexity science inspired guidance on how to use this data in a meaningful way.^85^

The benefits of ‘slow science’ are especially needed when we are dealing with complex systems. ‘Slowing down is about asserting the importance of contemplation, connectedness, fruition, and complexity. It gives meaning to letting research take the time: it needs to ripen and makes it easier to resist the pressure to be faster; it gives meaning to thinking about scholarship as a community, not a competition.^86^

## Conclusions

In short, complexity science provides a perspective that can be helpful when we research inherently complex and unpredictable systems, such as those we encounter in pediatrics. It can help us lessen the gap between evidence and practice by on the one hand improving our understanding of complex systems, and on the other hand by acknowledging the inherent unpredictability of complex systems and developing means to anticipate, adapt, learn and respond (Fig. 1).

It can give guidance in terms of asking better questions, choosing more appropriate and diverse methods, and drawing careful conclusions that our data can support. What is mostly required is so-called ‘negative capacity’, or the ‘ability to resist the urge to conclude’.^46^ There is discomfort in uncertainty. Complexity science asks us to resist that, to stay in uncomfortable uncertainty, to learn from it, and to expect the unexpected.^4,47,48^ It requires slow science, persistence, and curiosity. It encourages us to take time to answer the questions that really matter. And it underlines the need to stay humble about what we think we know.

We hope that this article will increase awareness of complexity and provides a starting point for clinical researchers in pediatrics to explore this perspective in their own research and their clinical practice.

## Data Availability

Data sharing not applicable to this article as no datasets were generated or analysed during the current study.
